# Efficacy of panniculectomy-combined surgery in superobese patients with endometrial cancer: A case report and literature review

**DOI:** 10.1016/j.ijscr.2020.05.066

**Published:** 2020-06-06

**Authors:** Masayo Okawa, Hiroaki Komatsu, Daiken Osaku, Mayumi Sawada, Akiko Kudoh, Ruri Shimogai, Jun Chikumi, Shinya Sato, Tetsuro Oishi, Tasuku Harada

**Affiliations:** Department of Obstetrics and Gynecology, Tottori University School of Medicine, 36-1 Nishicho, Yonago, Tottori, 683-5804, Japan

**Keywords:** Obesity, Panniculectomy, Endometrial cancer

## Abstract

**Introduction:**

Panniculectomy is regarded as an effective approach in highly obese patients with endometrial cancer to improve surgical access or space of the surgical field.

**Presentation of case:**

A 66-year-old nulliparous woman was brought in from another hospital to our institute for newly diagnosed endometrial carcinoma. The patient was 158 cm in height and weighed 135.8 kg during the first visit to our hospital. Her body mass Index (BMI) was 54.4 kg/m^2^. Unfortunately, the patient developed an umbilical hernia and strangulation before the surgery. Emergency partial ileal resection and simple closure of the umbilical hernia were performed. We were finally able to perform simple hysterectomy, and bilateral salpingo-oophorectomy with panniculectomy. Her weight at the time of surgery was 115.5 kg which had been reduced by 20 kg from the time she was first brought in, and the BMI decreased from 54.4 to 45.3 kg/m^2^. The patient underwent follow-up without adjuvant therapy since she was in the low-risk group, and showed no signs of recurrence 12 months after surgery.

**Discussion:**

There are various risks associated with surgery in highly obese patients. Diabetes mellitus, hypertension and smoking are reported to be risk factors of wound complication in panniculectomy, and it was reported that diabetes mellitus was an independent risk factor.

**Conclusion:**

The combination of panniculectomy was considered as an effective approach to perform safe surgery for obese patients.

## Highlights

• Incidence of endometrial cancer has shown a remarkable increase globally.
• Case of an obese patient who underwent panniculectomy combined surgical staging.
• Combination of panniculectomy was effective for surgery in obese patients.

## 1 Introduction

This case report has been reported in accordance with the SCARE criteria [1].

The incidence of endometrial cancer has shown a remarkable increase globally, in recent times. Although the incidence of endometrial cancer in Japan is lower than that in Europe or in the United States, it has been increasing. The number of patients with endometrial cancer in Japan was approximately 1000 in the 1970s; however, the number had increased to 14,763 in 2011. In recent times, it has become the most common gynecological malignancy in Japan.

Obesity is more strongly associated with the development of endometrial cancer than with any other cancer type [2]. The incidence of obesity in Japan was reported to be 31.3 % for men and 20.6 % for women aged 20 years and above, according to the “Summary of National Health and Nutrition Survey Results” in 2016. In particular, obesity increased with age in women.

An increase in the average life span and the incidence of obesity have increased the incidence of endometrial cancer. It is expected to further rise in the future. Actually, the opportunity to treat obese patients with endometrial cancer. However, in highly obese patients, performing laparotomy is often difficult due to the thickness of the abdominal wall. In addition, it has been reported that obesity increases the perioperative complications and significantly increases the duration of surgery and hospitalization.

Panniculectomy involves excising the excess skin and underlying adipose layer that forms the overhanging pannus. Panniculectomy is regarded as an effective approach in highly obese patients with endometrial cancer to improve surgical access or space of the surgical field. We reviewed previous literature to evaluate the outcomes of surgical procedures in superobese patients with endometrial cancer. We report here, the case of a superobese female patient who underwent panniculectomy combined surgical staging in our hospital.

## 2 Presentation of the case

A 66-year-old nulliparous woman was brought in from another hospital to our institute in February 2018 for newly diagnosed endometrial carcinoma. It was incidentally detected due to abnormal endometrial thickness on magnetic resonance imaging (MRI), which was performed to check the progress of a submucosal bladder tumor. The patient reported no prior history of a gynecologic disorder and had no symptoms of gynecologic disease. Pap smear was negative for malignancy. The result of endometrial cytology suggested endometrial cancer and endometrial biopsy revealed a well-differentiated endometrioid carcinoma.

**Fig. 1:**
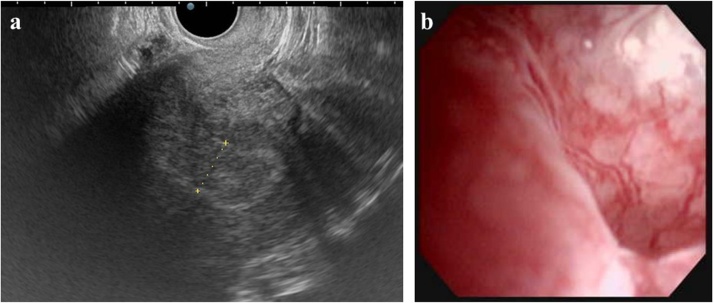
(a) Transvaginal ultrasonography: 20 mm of endometrium thickness (broken line). (b) Hysteroscopy: Tumor lesion with entire circumference of atypical vessel in corpus uteri.

The patient was 158 cm in height and weighed 135.8 kg during the first visit to our hospital. Her body mass Index (BMI) was 54.4 kg/m^2^. Ultrasonography showed a 20 mm endometrial thickening and multiple nodules of uterine myoma. Hysteroscopy revealed a protruding lesion with atypical vessels in the entire uterine cavity (Fig. 1a, b).

**Fig. 2:**
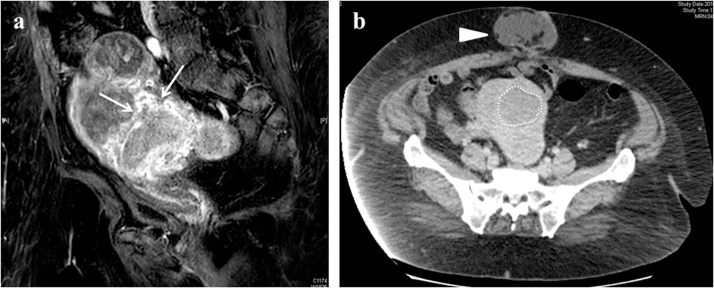
(a) Pelvic MRI (sagittal section of dynamic MRI) disruption of subendometrial enhancement in from uterine anterior wall to the fundus (white arrow). (b) Contrast-enhanced CT (horizontal section) tumor lesion in uteri (white broken line) and umbilical hernia (white triangle).

An examination of the sub-endometrial enhancement with dynamic MRI aroused the suspicion of a myometrial invasion in the anterior wall of the uterus, less than 1/2 thickness (Fig. 2a). A computed tomography examination showed an umbilical hernia and no distant metastasis or lymph node involvement (Fig. 2b). The patient was diagnosed as endometrial cancer stage 1A which is considered a low-risk group. We planned a simple hysterectomy and bilateral oophorectomy without lymphadenectomy. Finally, we planned a transabdominal total hysterectomy with panniculectomy for this patient.

The patient was admitted to the hospital to reduce her weight before the surgery because of the high risk of complications caused by anesthesia during the surgery and the difficulty of managing the diet at home by herself. With diet control her weight reduced from 135.8 kg to 115.5 kg after 4 weeks. Though we decided to perform the surgery in May 2018, we had to postpone it because she developed bronchitis.

**Fig. 3:**
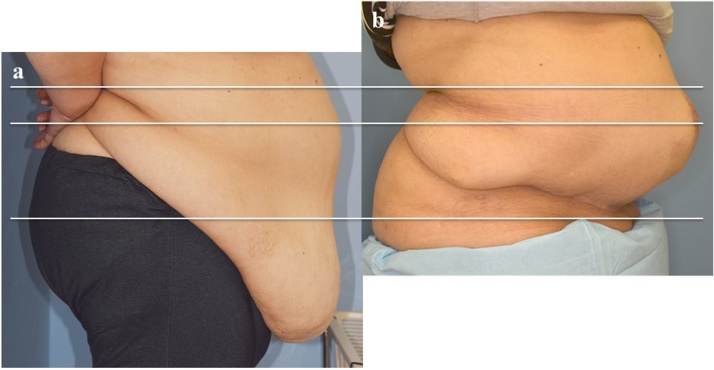
(a) Before diet: body weight: 132.2kg, BMI: 52.9 kg/m^2^. (b) 8 months after surgery: body weight: 117.0kg, BMI: 46.8 kg/m^2^.

After the patient's recovery, we scheduled a surgery in July 2018. Unfortunately, the patient developed an umbilical hernia and strangulation before the surgery. Emergency partial ileal resection and simple closure of the umbilical hernia were performed by the general surgeon at our hospital. In August 2018, we were finally able to perform simple hysterectomy, and bilateral salpingo-oophorectomy with panniculectomy. Her weight at the time of surgery was 115.5 kg which had been reduced by 20 kg from the time she was first brought in, and the BMI decreased from 54.4 to 45.3 kg/m^2^ (Fig. 3a, b).

**Fig. 4:**
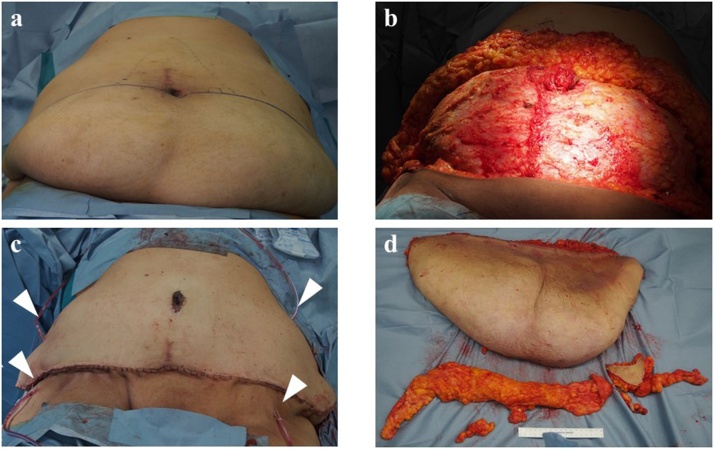
Procedure of panniculectomy. (a) Incised line was designed such that cranial line was at the umbilicus and the caudal end was at the superior anterior iliac spine. (b) Extra skin and fat on the fascia were excised. (c) After excision of the extra skin, umbilication was done. Four JVAC drains were retained on the fascia at the time of closing. (d) Removed skin and fat was 6,870 g in weight.

Initially a plastic surgeon performed the panniculectomy. A skin-incision was made with the cranial end of the incision at the umbilicus and the caudal end of the incision at the anterior superior iliac spine. The excess skin and fat like dog ear, which weighed 6.870 kg together, were removed above the abdominal fascia and navel-plasty was performed (Fig. 4). Thereafter a simple total hysterectomy and bilateral salpingo-oophorectomy was performed. The uterus had enlarged to more than fist size due multiple uterine fibroids. The rectum was adhered to the posterior uterine wall. All procedures were performed using the same retractor without any difficulty. The surgical time for panniculectomy was 3 h and 12 min, and that for hysterectomy was 2 h and 29 min. Total volume of blood loss was 420 mL. At the time of closure, four subcutaneous drains were placed on the fascia to prevent dead space formation.

After the surgery, low molecular weight heparin was administered to prevent deep vein thrombosis. On the 14th day after surgery, the navel wound showed local infection and required oral minocycline and washing out her wound by herself. To treat the wound infection, the hospital stay was extended by several days. The patient was discharged on the 26th after hysterectomy. The weight and BMI at discharge were 105.9 kg and 41.7 kg/m^2^ respectively.

**Fig. 5:**
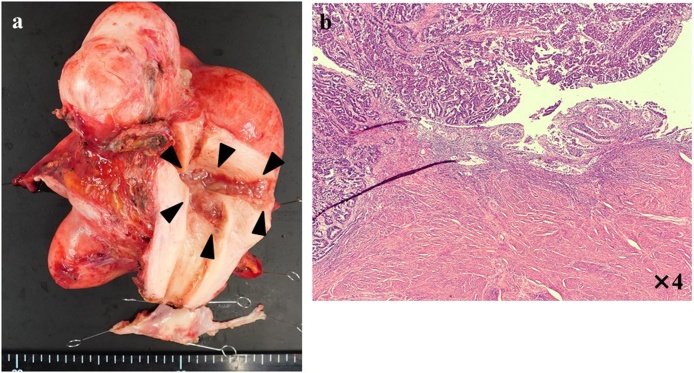
(a) Surgical specimen (uteri and both of adnexa): tumor lesion in endometrial cavity (black triangle). (b) Histopathologic examination: dense hyperplasia of atypical endometrial duct.

The histopathological diagnosis was endometrial cancer stage IA, pT1aN0M0, grade 1 endometrioid carcinoma (Fig. 5a, b). The patient underwent follow-up without adjuvant therapy since she was in the low-risk group, and showed no signs of recurrence 12 months after surgery.

## 3 Discussion

This was the case of a severely obese patient with endometrial cancer. We achieved perioperative management without any difficulty in this patient who underwent transabdominal total hysterectomy with panniculectomy.

There are various risks associated with surgery in highly obese patients. In the management of general anesthesia, there are concerns related to airway management, hypoxemia during artificial respiration, changes in the circulatory dynamics, regulating the doses of anesthetics, intraoperative awakening, respiratory failure and hypoxemia after surgery, and occurrence of thromboembolism [3]. Moreover, the duration of surgery might be prolonged due to difficulty in obtaining an adequate operative field.

Although laparoscopy is superior to laparotomy once an adequate surgical view is achieved, it increases the risk of hypercapnia and hypoxemia during surgery because of the effect of anesthetic gases on the respiratory and circulatory systems. Furthermore, in laparoscopic surgeries for gynecological diseases, the Trendelenburg’s position can greatly affect the cardiopulmonary dynamics, and further increase the risk of hypercapnia and hypoxemia during surgery [4]．It is necessary to consider these risks before deciding on the surgical indication and technique. Especially, highly obese patients with BMI more than 35 the reported rate of conversion from laparoscopy to laparotomy, and increases to 8.6 % in those whose BMI exceeds 40 [5,6]．

In this case, we initially planned laparoscopy, but finally chose laparotomy. Since the patient’s preoperative BMI was 45.3 kg/m^2^, management of intraoperative anesthesia was predicted to be difficult. With laparotomy, we successfully maintained stable intraoperative and postoperative cardiopulmonary dynamics.

Massenburg et al. reported that the combination of hysterectomy and panniculectomy reduced surgical complications, surgical time, time under anesthesia and duration of hospitalization [7]．Hardy et al. similarly reported that the combination with panniculectomy reduced the incidence rate of intraoperative complications and postoperative wound complications, without increasing the duration of surgery and blood loss [8]．

The incidence of intraoperative complications in panniculectomy was reported to be 31–76%, including hematoma 5.7 %, infection 4.5 %, skin necrosis 2.7 %, wound dehiscence 1.3 %, and deep venous thrombosis (DVT) 0.2 % [9,10].

Diabetes mellitus, hypertension and smoking are reported to be risk factors of wound complication in panniculectomy, and it was reported that diabetes mellitus was an independent risk factor [11,12]. It was reported that the incidence of wound infection increased when the surgery was performed in summer rather than in winter [13]．This patient developed wound infection after surgery and required wound cleaning and antibiotics. In this case, the comorbidities of hypertension and diabetes mellitus, the postponement of the surgery twice, and performing the surgery in summer, might have contributed to the wound infection. It was reported that prophylactic administration of antibiotics did not reduce wound infections in obese patients with panniculectomy [14]. Long-term administration of antibiotics for prevention infection is not recommended.

Because of the excision extent and the invasion of panniculectomy, postoperative pain control is difficult. Epidural anesthesia, rectus sheath block (RSB), transversus abdominal plane block (TAPB) administered by anesthesiologists, and subcutaneous infiltration (SCI) administered by surgeons are used for eliminating pain. In a report comparing the analgesic efficacy of different pain management modalities in panniculectomy, it was seen that with TAPB the pain time was significantly longer and the required dosage of morphine was lower than in RSB and SCI [15,16]．In this case, the patient was provided only epidural anesthesia and regular intravenous drips of acetaminophen for postoperative pain control, and she was able to achieve early ambulation. However, laparoscopy can reduce wound pain compared to laparotomy, and is thus useful in obese patients. If laparoscopy was possible in this case, it should have been performed minimally invasive surgery.

## 4 Conclusion

The combination of panniculectomy with hysterectomy was considered as an effective approach to perform safe surgery for obese patients. After achieving complete oncological cure, it is important to select appropriate surgical techniques and provide appropriate perioperative management for reducing the perioperative complications and improving the quality of life of patients.

## Declaration of Competing Interest

The authors declare that they have no conflict of interest.

## Funding

This research did not receive any specific grant from funding agencies in the public, commercial, or not-for-profit sectors.

## Ethical approval

The present study was approved by the Institutional Review Board of Tottori University Hospital.

## Consent

All patients provided written informed consent before the collection of specimens according to the institutional guidelines.

## Author contribution

D.O devised the project, the main conceptual ideas, and proof outline. H.K and M.O worked out almost all the technical details and performed numerical calculations for the suggested experiment. H.K, M.O, D.O, S.S wrote the manuscript. All authors provided critical feedback and helped shape the research, analysis, and manuscript.

## Registration of research studies

1 Name of the registry:
2 Unique identifying number or registration ID:
3 Hyperlink to your specific registration (must be publicly accessible and will be checked):

## Guarantor

Hiroaki Komatsu.

Tasuku Harada.

## Provenance and peer review

Not commissioned, externally peer-reviewed.
